# Laparoscopic primary suture repair of sigmoid perforation due to a chicken bone: a case report

**DOI:** 10.1093/jscr/rjaf511

**Published:** 2025-07-14

**Authors:** Pia Borgas, Michael F Bath, Ibrahim Jama, Charalampos Theodoropoulos, Subramaniam Guru Naidu

**Affiliations:** North Middlesex University Hospital, Royal Free London NHS Foundation Trust, Sterling Way, London, N18 1QX, United Kingdom; North Middlesex University Hospital, Royal Free London NHS Foundation Trust, Sterling Way, London, N18 1QX, United Kingdom; Department of Engineering, University of Cambridge, Trumpington Street, Cambridge, CB2 1PZ, United Kingdom; Barnet & Chase Farm Hospital, Royal Free London NHS Foundation Trust, 127 The Ridgeway, London, EN2 8JL, United Kingdom; Barnet & Chase Farm Hospital, Royal Free London NHS Foundation Trust, 127 The Ridgeway, London, EN2 8JL, United Kingdom; North Middlesex University Hospital, Royal Free London NHS Foundation Trust, Sterling Way, London, N18 1QX, United Kingdom; Barnet & Chase Farm Hospital, Royal Free London NHS Foundation Trust, 127 The Ridgeway, London, EN2 8JL, United Kingdom

**Keywords:** emergency surgery, colorectal, perforation, foreign body

## Abstract

Sigmoid colon perforation due to foreign body ingestion is a rare and potentially life-threatening condition. Whilst sigmoid perforation is often managed through surgical intervention, no guidelines exist regarding use of minimally invasive approaches in foreign body ingestion. We report the case of a 54-year-old male who presented with acute abdominal pain and localized peritonitis following the accidental ingestion of a chicken bone. Imaging revealed localized free intraperitoneal air and following laparoscopic exploration, a sigmoid perforation secondary to a chicken bone was identified. Laparoscopic primary suture repair was successfully carried out, with the patient making a full and uneventful recovery, being discharged on post-operative Day 9. The case highlights the feasibility and safety of laparoscopic primary suture repair in select cases of sigmoid colon perforation caused by an ingested foreign body. This should promote further research into this surgical case management.

## Introduction

Colonic perforation is a potentially life-threatening surgical emergency requiring prompt diagnosis and intervention. Whilst the most common cause, in high-income countries, is diverticular disease, other common aetiologies include malignancy, inflammatory bowel disease, and iatrogenic injury. Traumatic and foreign body-induced perforations of the colon are rare, but can occur due to either accidentally ingested objects, such as fish or chicken bones, or inserted foreign bodies [1, 2].

The complications of colonic perforation primarily arise from faecal contamination of the peritoneal cavity, which can lead to faeculent peritonitis, septic shock, and multi-organ failure if left untreated [3]. Whilst the standard surgical management in the majority of cases is with sigmoid resection, particularly in cases of diffuse peritonitis or haemodynamic instability [3], iatrogenic perforations, particularly those occurring during colonoscopy, have been increasingly managed through minimally invasive means; indeed, many iatrogenic perforations may be amenable to endoscopic closure if identified early [4]. However, the gold-standard surgical management in foreign body perforation remains contested and laparoscopic repair remains underutilized in the perforated sigmoid disease due to concerns regarding intra-abdominal contamination and technical demands of intracorporeal suturing.

Herein, we present a unique case of sigmoid perforation secondary to an ingested chicken bone, successfully managed with laparoscopic primary suture repair. To our knowledge, this is the first reported case of laparoscopic primary closure of a non-iatrogenic sigmoid perforation in a contaminated bowel.

## Case presentation

A 54-year-old male, who worked as a chef, presented to the emergency department with a 2-day history of worsening left iliac fossa (LIF) pain. The pain was stabbing in nature, radiating intermittently to the upper abdomen and back, and exacerbated by movement and lying flat. He reported associated fatigue and diarrhoea, but denied any nausea, vomiting, fever, or rectal bleeding. He had no recollection of any specific foreign body ingestion, although he had consumed takeaway food 3 days prior.

His past medical history included coronary artery disease, for which he had three stents placed 12 years ago, and for which he took ramipril, atorvastatin, aspirin, and bisoprolol. He was an ex-heavy smoker (40 pack-years, quit 10 years prior) with regular alcohol intake and had an American Society of Anesthesiologists score of 3.

On initial examination, he was alert and orientated and was hemodynamically stable on arrival. Abdominal examination revealed marked tenderness in the LIF with guarding and percussion tenderness, without any generalized peritoneal rigidity. Laboratory tests showed a leucocytosis (white cell count 15 × 10^9^/l, neutrophils 13 × 10^9^/l) and elevated C-reactive protein (53 mg/l), with normal renal function and lactate.

**Figure 1:**
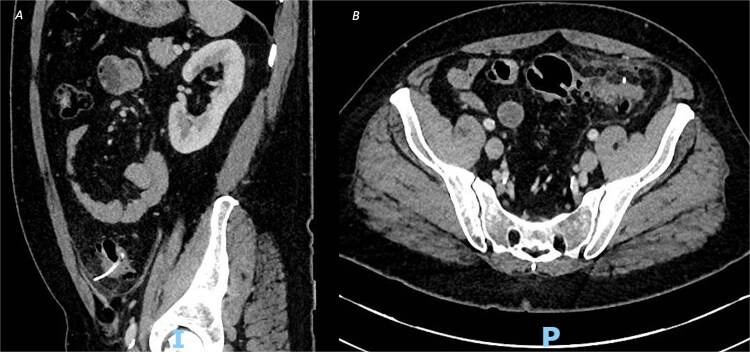
CT of the abdomen and pelvic with contrast, demonstrating an ingested foreign body with localized perforation and pericolic inflammatory changes, in both (A) sagittal view and (B) axial view.

An urgent contrast-enhanced computed tomography (CT) abdomen–pelvis was performed, which revealed a 3.7-cm linear hyperdense foreign body, consistent with a fish or chicken bone, penetrating the wall of the sigmoid colon with associated surrounding pericolic fat stranding and small amount of localized pericolic air denoting small perforation (Fig. 1); there was no evidence of abscess formation, or distant free air.

A decision was made to proceed with an exploratory laparoscopy and attempted primary suture repair of the defect. The patient was made aware during the consenting process of the possibility of laparotomy, bowel resection, and stoma, depending on the findings at laparoscopy. During laparoscopy, the site of perforation in the sigmoid colon was identified, at the site of the foreign body penetration by the chicken bone. Following careful removal of overlying omentum, the 3 × 40 mm bone was successfully removed laparoscopically and the 3 mm diameter perforation site in the sigmoid colon identified. The defect was repaired primarily with three interrupted 3-0 Polydioxanone suture (PDS). A neighbouring epiploic appendage of the sigmoid colon was sutured over the site of the repaired defect for extra support. Finally, the omentum was placed over the perforation site, but not sutured to it due to the risk of creating an iatrogenic omental defect through which the bowel could herniate in the future. No conversion to open or stoma formation was required, and two intra-abdominal drains were placed.

**Figure 2:**
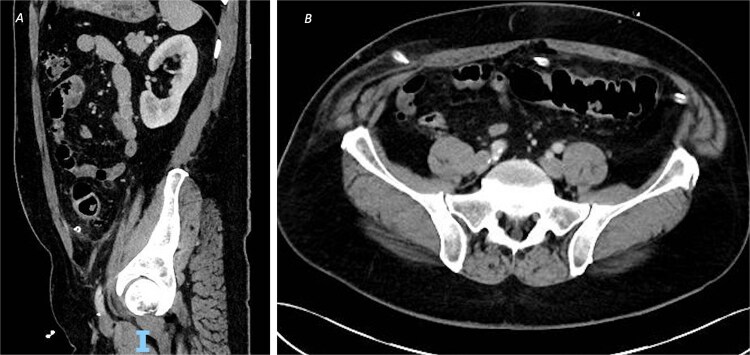
CT of the abdomen and pelvic with contrast on post-operative Day 8 demonstrating resolution of the inflammatory changes with no evidence of leak or abscess formation, from (A) sagittal view and (B) axial view.

Post-operatively, the patient made a good recovery. As per local policy, he remained on intravenous antibiotics until discharge. In the immediate post-operative period, he was kept nil-by-mouth for 2 days and received total parenteral nutrition, before having his dietary intake increased after he passed flatus and opened his bowels. The patient was tolerating a normal diet by post-operative Day 5, with serial inflammatory markers also improving. A repeat CT scan on post-operative Day 8 confirmed resolution of the inflammatory changes with no evidence of leak or abscess formation (Fig. 2), and his intra-abdominal drains were subsequently removed.

The patient was discharged home on post-operative Day 9 and was back to normal activities at his follow-up in clinic 2 weeks later.

## Discussion

We report the first documented case of laparoscopic repair of a sigmoid perforation secondary to a foreign body. Primary suture repair of sigmoid perforations has traditionally been limited to controlled settings, particularly iatrogenic perforations during endoscopic procedures [5–8]. However, foreign body perforation often presents in a similar patient cohort, with minimal localized perforation, and in clinically stable patients. This case report demonstrates that laparoscopic repair of colonic perforation through foreign body ingestion should be considered in appropriate cases.

Historically, the gold-standard treatment for colonic perforation has been resection and primary anastomosis or Hartmann’s procedure, particularly in the presence of contamination or sepsis [9]. However, recent advances in laparoscopic techniques have enabled minimally invasive approaches to be successfully employed in select patients. Indeed, several studies have reported successful direct closure of sigmoid perforation following to endoscopic intervention [5–8]. Patients who have had an endoscopic perforation often have had previous bowel preparation, leading to minimal faecal contamination; however, no previous high-quality studies have looked at the feasibility of primary closure within a contaminated setting, despite these clear parallels.

This case represents a unique instance of laparoscopic primary closure of a non-iatrogenic sigmoid perforation, managed without diversion in the setting of mild peritoneal contamination and a small defect size. Key factors that influenced the decision to proceed with suture repair instead of resection and possible stoma included focal perforation without gross faecal contamination, no haemodynamic instability in an otherwise well patient, and a relatively small defect, at 3 mm in diameter. Laparoscopic management is known to offer several benefit over traditional open surgery, such as lower rates of surgical site infection, decreased post-operative pain, shorter hospital stays, and reduced risks associated of incisional hernias and/or adhesive bowel obstruction [10]. There is also less risk of the patient ending up with a stoma and associated stoma complications. Therefore, appropriate patient selection in this case has led to optimal clinical outcomes.

Despite these advantages, laparoscopic suture repair presents several technical challenges. Intracorporeal suturing in the presence of friable or inflamed tissue increases the risk of suture dehiscence and subsequent leak [11], therefore patients should be selected carefully to avoid these risks. Additionally, sutures should be tension-free with adequate serosal apposition and reinforcement techniques should be considered such as omental patching or epiploic appendage coverage, as these have been described in similar cases previously and applied successfully in this patient [12, 13].

## Conclusion

This case highlights the feasibility and advantages of laparoscopic primary suture repair in select cases of sigmoid perforation, offering the advantage of minimally invasive technique whilst preserving bowel continuity and avoiding the need for a stoma. This challenges traditional paradigms, where resection is often the default approach for colonic perforations, and supports an individualized approach based on intraoperative findings. Given the increasing availability of advanced laparoscopic skills, this technique should be considered in select cases to reduce the morbidity associated with bowel resection and stoma formation. Further research including case series and comparative studies is needed to better define selection criteria for primary suture repair and establish guidelines for its wider adoption in colorectal surgery.
